# Transcriptional control in the prereplicative phase of T4 development

**DOI:** 10.1186/1743-422X-7-289

**Published:** 2010-10-28

**Authors:** Deborah M Hinton

**Affiliations:** 1 Laboratory of Molecular and Cellular Biology, National Institute of Diabetes and Digestive and Kidney Diseases, National Institutes of Health, (Building 8, Room 2A-13) Bethesda, MD (20892-0830) USA

## Abstract

Control of transcription is crucial for correct gene expression and orderly development. For many years, bacteriophage T4 has provided a simple model system to investigate mechanisms that regulate this process. Development of T4 requires the transcription of early, middle and late RNAs. Because T4 does not encode its own RNA polymerase, it must redirect the polymerase of its host, *E. coli*, to the correct class of genes at the correct time. T4 accomplishes this through the action of phage-encoded factors. Here I review recent studies investigating the transcription of T4 prereplicative genes, which are expressed as early and middle transcripts. Early RNAs are generated immediately after infection from T4 promoters that contain excellent recognition sequences for host polymerase. Consequently, the early promoters compete extremely well with host promoters for the available polymerase. T4 early promoter activity is further enhanced by the action of the T4 Alt protein, a component of the phage head that is injected into *E. coli *along with the phage DNA. Alt modifies Arg265 on one of the two α subunits of RNA polymerase. Although work with host promoters predicts that this modification should decrease promoter activity, transcription from some T4 early promoters increases when RNA polymerase is modified by Alt. Transcription of T4 middle genes begins about 1 minute after infection and proceeds by two pathways: 1) extension of early transcripts into downstream middle genes and 2) activation of T4 middle promoters through a process called sigma appropriation. In this activation, the T4 co-activator AsiA binds to Region 4 of σ^70^, the specificity subunit of RNA polymerase. This binding dramatically remodels this portion of σ^70^, which then allows the T4 activator MotA to also interact with σ^70^. In addition, AsiA restructuring of σ^70 ^prevents Region 4 from forming its normal contacts with the -35 region of promoter DNA, which in turn allows MotA to interact with its DNA binding site, a MotA box, centered at the -30 region of middle promoter DNA. T4 sigma appropriation reveals how a specific domain within RNA polymerase can be remolded and then exploited to alter promoter specificity.

## Background

Expression of the T4 genome is a highly regulated and elegant process that begins immediately after infection of the host. Major control of this expression occurs at the level of transcription. T4 does not encode its own RNA polymerase (RNAP), but instead encodes multiple factors, which serve to change the specificity of polymerase as infection proceeds. These changes correlate with the temporal regulation of three classes of transcription: early, middle, and late. Early and middle RNA is detected prereplicatively [previously reviewed in [1-6]], while late transcription is concurrent with T4 replication and discussed in another chapter. T4 early transcripts are generated from early promoters (Pe), which are active immediately after infection. Early RNA is detected even in the presence of chloramphenicol, an antibiotic that prevents protein synthesis. In contrast, T4 middle transcripts are generated about 1 minute after infection at 37°C and require phage protein synthesis. Middle RNA is synthesized in two ways: 1) activation of middle promoters (Pm) and 2) extension of Pe transcripts from early genes into downstream middle genes.

This review focuses on investigations of T4 early and middle transcription since those detailed in the last T4 book [1,5]. At the time of that publication, early and middle transcripts had been extensively characterized, but the mechanisms underlying their synthesis were just emerging. In particular, *in vitro *experiments had just demonstrated that activation of middle promoters requires a T4-modified RNAP and the T4 activator MotA [7,8]. Subsequent work has identified the needed RNAP modification as the tight binding of a 10 kDa protein, AsiA, to the σ^70 ^subunit of RNAP [9-13]. In addition, a wealth of structural and biochemical information about *E. coli *RNAP [reviewed in [14-16]], MotA, and AsiA [reviewed in [2]] has now become available. As detailed below, we now have a much more mechanistic understanding of the process of prereplicative T4 transcription. To understand this process, we first start with a review of the host transcriptional machinery and RNAP.

### The *E. coli *transcriptional machinery

*E. coli *RNAP holoenzyme, like all bacterial RNAPs, is composed of a core of subunits (β, β',α_1_, α_2_, and ω), which contains the active site for RNA synthesis, and a specificity factor, σ, which recognizes promoters within the DNA and sets the start site for transcription. The primary σ, σ^70 ^in *E. coli*, is used during exponential growth; alternate σ factors direct transcription of genes needed during different growth conditions or times of stress [reviewed in [17-19]]. Sequence/function analyses of hundreds of σ factors have identified various regions and subregions of conservation. Most σ factors share similarity in Regions 2-4, the central through C-terminal portion of the protein, while primary σ factors also have a related N-terminal portion, Region 1.

**Figure 1:**
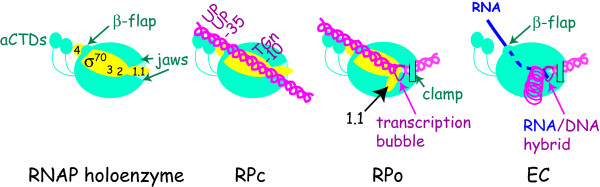
**RNAP holoenzyme and the interaction of RNAP with σ**^**70**^**-dependent promoters**. Structure-based cartoons (left to right) depict RNAP holoenzyme, RPc (closed complex), RPo (open complex), and EC (elongating complex) with σ^70 ^in yellow, core (β,β',α_2_, and ω) in turquoise, DNA in magenta, and RNA in purple. In holoenzyme, the positions of σ^70 ^Regions 1.1, 2, 3, and 4, the α-CTDs, the β-flap, and the β,β' jaws are identified. In RPc, contact can be made between RNAP and promoter dsDNA elements: two UP elements with each of the α-CTDs, the -35 element with σ^70 ^Region 4, TGn (positions -15 to -13) with σ^70 ^Region 3, and positions -12/-11 of the -10 element with σ^70 ^Region 2. σ^70 ^Region 1.1 lies in the downstream DNA channel formed by portions of β and β' and the β',β' jaws are open. In RPo, unwinding of the DNA and conformational changes within RNAP result in a sharp bend of the DNA into the active site with the formation of the transcription bubble surrounding the start of transcription, the interaction of σ^70 ^Region 2 with nontemplate ssDNA in the -10 element, movement of Region 1.1 from the downstream DNA channel, and contact between the downstream DNA and the β' clamp. In EC, σ^70 ^and the promoter DNA have been released. The newly synthesized RNA remains annealed to the DNA template in the RNA/DNA hybrid as the previously synthesized RNA is extruded through the RNA exit channel past the β-flap.

Recent structural information, together with previous and ongoing biochemical and genetic work [reviewed in [14,15,20,21]], has resulted in a biomolecular understanding of RNAP function and the process of transcription. Structures of holoenzyme, core, and portions of the primary σ of thermophilic bacteria with and without DNA [15,16,22-28], and structures of regions of *E. coli *σ^70 ^alone [29] and in a complex with other proteins [26,30] are now available. This work indicates that the interface between σ^70 ^and core within the RNAP holoenzyme is extensive (Figure 1). It includes contact between a portion of σ Region 2 and a coiled/coil domain composed of β, β', an interaction of σ^70 ^Region 1.1 within the "jaws" in the downstream DNA channel (where DNA downstream of the transcription start site will be located when RNAP binds the promoter), and an interaction between σ^70 ^Region 4 and a portion of the β subunit called the β-flap.

For transcription to begin, portions of RNAP must first recognize and bind to double-stranded (ds) DNA recognition elements present within promoter DNA (Figure 1) [reviewed in [20]]. Each of the C-terminal domains of the α subunits (α-CTDs) can interact with an UP element, A/T rich sequences present between positions -40 and -60. Portions of σ^70^, when present in RNAP, can interact with three different dsDNA elements. A helix-turn-helix, DNA binding motif in σ^70 ^Region 4 can bind to the -35 element, σ^70 ^Region 3 can bind to a -15TGn-13 sequence (TGn), and σ^70 ^subregion 2.4 can bind to positions -12/-11 of a -10 element. Recognition of the -35 element also requires contact between residues in σ^70 ^Region 4 and the β-flap in order to position σ^70 ^correctly for simultaneous contact of the -35 and the downstream elements. Typically, a promoter only needs to contain two of the three σ^70^-dependent elements for activity; thus, *E. coli *promoters can be loosely classified as -35/-10 (the major class), TGn/-10 (also called extended -10), or -35/TGn [reviewed in [20]].

The initial binding of RNAP to the dsDNA promoter elements usually results in an unstable, "closed" complex (RPc) (Figure 1). Creation of the stable, "open" complex (RPo) requires bending and unwinding of the DNA [31] and major conformational changes (isomerization) of the polymerase (Figure 1) [[32,33]; reviewed in [20]]. In RPo the unwinding of the DNA creates the transcription bubble from -11 to ~+3, exposing the single-stranded (ss) DNA template for transcription. Addition of ribonucleoside triphosphates (rNTPs) then results in the synthesis of RNA, which remains as a DNA/RNA hybrid for about 8-9 bp. Generation of longer RNA initiates extrusion of the RNA through the RNA exit channel formed by portions of β and β' within core. Since this channel includes the σ^70^-bound β-flap, it is thought that the passage of the RNA through the channel helps to release σ from core, facilitating promoter clearance. The resulting elongation complex, EC, contains core polymerase, the DNA template, and the synthesized RNA (Figure 1) [reviewed in [34]]. The EC moves rapidly along the DNA at about 50 nt/sec, although the complex can pause, depending on the sequence [35]. Termination of transcription occurs either at an intrinsic termination signal, a stem-loop (hairpin) structure followed by a U-rich sequence, or a Rho-dependent termination signal [reviewed in [36,37]]. The formation of the RNA hairpin by an intrinsic terminator sequence may facilitate termination by destabilizing the RNA/DNA hybrid. Rho-dependent termination is mediated through the interaction of Rho protein with a rut site (Rho utilization sequence), an unstructured, sometimes C-rich sequence that lies upstream of the termination site. After binding to the RNA, Rho uses ATP hydrolysis to translocate along the RNA, catching up with the EC at a pause site. Exactly how Rho disassociates a paused complex is not yet fully understood; the DNA:RNA helicase activity of Rho may provide a force to "push" RNAP off the DNA. Rho alone is sufficient for termination at some Rho-dependent termination sites. However, at other sites the termination process also needs the auxiliary *E. coli *proteins NusA and/or NusG [reviewed in [36].

When present in intergenic regions, rut sites are readily available to interact with Rho. However, when present in protein-coding regions, these sites can be masked by translating ribosomes. In this case, Rho termination is not observed unless the upstream gene is not translated, for example, when a mutation has generated a nonsense codon. In such a case, Rho-dependent termination can prevent transcription from extending into the downstream gene. Thus, in this situation, which is called polarity [38], expression of both the upstream mutated gene and the downstream gene is prevented.

### T4 early transcription

#### Early promoters

**Figure 2:**
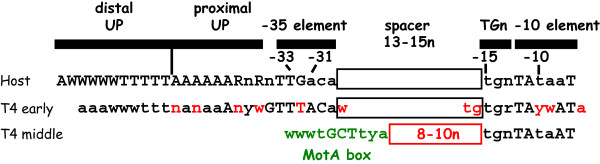
**Comparison of *E. coli *host, T4 early, and T4 middle promoter sequences**. Top, Sequences and positions of host promoter recognition elements for σ^70^-RNAP (UP, -35, TGn, -10) are shown [20,150]. Below, similar consensus sequences found in T4 early [4] and middle [91] promoters are in black and differences are in red; the MotA box consensus sequence in T4 middle promoters is in green. Spacer lengths between the TGn elements and the -35 elements (host and T4 early) or the MotA box are indicated. W = A or T; R = A or G; Y = C or T, n = any nucleotide; an uppercase letter represents a more highly conserved base.

T4 only infects exponentially growing *E. coli*, and transcription of T4 early genes begins immediately after infection. Thus, for an efficient infection, the phage must rapidly redirect the σ^70^-associated RNAP, which is actively engaged in transcription of the host genome, to the T4 early promoters. This immediate takeover is successful in part because most T4 early promoters contain excellent matches to the σ^70^-RNAP recognition elements (-35, TGn, and -10 elements) and to the α-CTD UP elements (Figure 2; for lists of T4 early promoter sequences, see [4,5]). However, sequence alignments of T4 early promoters reveal additional regions of consensus, suggesting that they contain other bits of information that can optimize the interaction of host RNAP with the promoter elements. Consequently, unlike most host promoters that belong to the -35/-10, TGn/-10 or -35/TGn class, T4 early promoters can be described as "über" UP/-35/TGn/-10 promoters. Indeed, most T4 early promoters compete extremely well with the host promoters for the available RNAP [39] and are similar to other very strong phage promoters, such as T7 P_A1 _and λ P_L_.

#### The T4 Alt protein

Besides the sheer strength of its early promoters, T4 has another strategy, the Alt protein, to establish transcriptional dominance [[40-43], reviewed in [1,4]]. Alt, a mono-ADP-ribosyltransferase, ADP-ribosylates a specific residue, Arg265, on one of the two α subunits of RNAP. In addition, Alt modifies a fraction of other host proteins, including the other RNAP subunits and host proteins involved in translation and cell metabolism. Alt is an internal phage head protein that is injected with the phage DNA. Consequently, Alt modification occurs immediately after infection and does not require phage protein synthesis. Each α subunit is distinct (one α interacts with β while the other interacts with β') and Alt modification is thought to specifically target a particular α, although which particular α is not known.

What is the purpose of Alt modification? The major Alt target, α Arg265, has been shown to be crucial for the interaction of an α-CTD with a promoter UP element [44-46] and with some host activators, including c-AMP receptor protein (CRP), a global regulator of *E. coli *[46,47]. Thus, an obvious hypothesis is that Alt simply impairs host promoters that either need these activators or are enhanced by α-CTD/UP element interaction. However, overexpression of Alt from a plasmid does not affect *E. coli *growth [40], and general transcription of *E. coli *DNA *in vitro *is not impaired when using Alt-modified RNAP [48]. Instead, it appears that Alt-modification is helpful because it increases the activity of certain T4 early promoters. This 2-fold enhancement of activity has been observed both *in vivo *[40,49] and *in vitro *[48]. How Alt-modification stimulates particular early promoters is not known, but it is clear that it is not simply due to their general strength. Other strong promoters, such as P_tac_, T7 P_A1 _and P_A2_, T5 P_207_, and even some of the T4 early promoters, are unaffected when using Alt-modified RNAP [49]. Alt-mediated stimulation of a promoter is also not dependent on specific σ^70^-dependent elements (-35, TGn, and -10 elements); some promoters with identical sequences in these regions are stimulated by Alt while others are not [49]. A comprehensive mutational analysis of the T4 early promoter P_8.1 _and P_tac _reveals that there is not a single, specific promoter position(s) responsible for the Alt effect. This result suggests that the mechanism of Alt stimulation may involve cross-talk between RNAP and more than one promoter region [50] or that ADP-ribosylation of α Arg265 is a secondary, less significant activity of Alt and additional work on the importance of this injected enzyme is needed.

#### Continuing early strategies for T4 domination

Because T4 promoters are so efficient at out-competing those of the host, a burst of immediate early transcription occurs within the first minute of infection. From this transcription follows a wave of early products that continue the phage takeover of the host transcriptional machinery. One such product is the T4 Alc protein, a transcription terminator that is specific for dC-containing DNA, that is, DNA that contains unmodified cytosines. Consequently, Alc terminates transcription from host DNA without affecting transcription from T4 DNA, whose cytosines are hydroxymethylated and glucosylated [[51,52]; reviewed in [1,4]]. Alc directs RNAP to terminate at multiple, frequent, and discrete sites along dC-containing DNA. The mechanism of Alc is not known. Unlike other terminating factors, Alc does not appear to interact with either RNA or DNA, and decreasing the rate of RNA synthesis or RNAP pausing near an Alc termination site actually impairs Alc termination [51]. Mutations within an N-terminal region of the β subunit of RNAP, a region that is not essential for *E. coli *(dispensable region I), prevent Alc -mediated termination, suggesting that an interaction site for Alc may reside in this region [52].

T4 also encodes two other ADP-ribosylating enzymes, ModA and ModB, as early products. Like Alt, ModA modifies Arg265 of RNAP α [[53,48]; reviewed in [1,4]]. However, unlike Alt, ModA almost exclusively targets the RNAP α subunits. In addition, ModA modifies both α subunits so there is no asymmetry to ModA modification. Synthesis of ModA is highly toxic to *E. coli*. *In vitro*, ModA-modified RNAP is unable to interact with UP elements or to interact with CRP [cited in [40]] and is less active than unmodified RNAP when using either *E. coli *or T4 DNA [48]. Thus, it has been suggested that ModA helps to diminish both host and T4 early promoter activity, reprogramming the transcriptional machinery for the coming wave of middle transcription [48]. However, a deletion of the *modA *gene does not affect the rapid decrease in early transcription or the decrease in the synthesis of early gene products, which begins about 3 minutes post-infection [54]. This result suggests that the phage employs other as yet unknown strategies to stop transcription from early promoters. ModB, the other early ADP-ribosylating enzyme, targets host translation factors, the ribosomal protein S30 and trigger factor, which presumably helps to facilitate T4 translation [43].

Finally, many of the early transcripts include genes of unknown function and come from regions of the T4 genome that are not essential for infection of wild type (wt) *E. coli *under normal laboratory conditions. Presumably, these genes encode phage factors that are useful under specific growth conditions or in certain strains. Whether any of these gene products aid T4 in its takeover of the host transcriptional machinery is not known.

#### The switch to middle transcription

Within a minute of infection at 37°C, some of the T4 early products mediate the transition from early to middle gene expression. As detailed below, the MotA activator and AsiA co-activator are important partners in this transition, since they direct RNAP to transcribe from middle promoters. In addition, the ComC-α protein, described later, may also have a role in the extension of early RNAs into downstream middle genes or the stability of such transcripts once they are formed.

As middle transcription begins, certain early RNAs decay rapidly after their initial burst of transcription. This arises from the activity of the early gene product RegB, an endoribonuclease, which specifically targets some T4 early mRNAs. For the mRNAs of MotA and RegB itself, a RegB cleavage site lies within the Shine-Dalgarno sequence; for ComC-α mRNA, the site is within AU-rich sequences upstream and downstream of this sequence [55]. The mechanism by which RegB recognizes and chooses the specific cleavage site is not yet known.

The onset of T4 middle transcription also finishes the process of eliminating host transcription by simply removing the host DNA template for RNAP. T4-encoded nucleases, primarily EndoII encoded by *denA *and EndoIV encoded by *denB*, selectively degrade the dC-containing host DNA ([56,57] and references therein). Thus, a few minutes after infection, there is essentially no host DNA to transcribe.

### Transcription of middle genes from T4 middle promoters

#### Middle promoters

Middle genes primarily encode proteins needed for replication, recombination, and nucleotide metabolism; various T4-encoded tRNAs; and transcription factors that program the switch from middle to late promoter activation. Middle RNAs arise by 2 pathways: extension of early transcription into middle genes (discussed later) and the activation of T4 middle promoters by a process called σ appropriation [2]). To date, nearly 60 middle promoters have been identified (Table 1). Unlike early promoters, T4 middle promoters contain a host element, the σ^70^-dependent -10 sequence, and a phage element, a MotA box, which is centered at -30 and replaces the σ^70^-dependent -35 element present in T4 early promoters and most host promoters (Figure 2). In addition, about half of the middle promoters also contain TGn, the extended -10 sequence. Activation of the phage middle promoters requires the concerted effort of two T4 early products, AsiA and MotA.

**Table 1:** Positions of identified T4 middle promoters

Middle Promoter	Start site	Reference
PrIIB2	122	[99,141,142]
PrIIB1	377	[99,141,142]
PrIIA	2263	[141,142]
P39	5349	[99]
Pdex.2	10058	[91]
Pdda.1	11138	[91]
P56/69	16813	[99]
Pdam	17617	[91]
P61	19122	[100]
PuvsX	23752	[7]
PsegA	24460	[7]
P42	26320	[100]
P43	29933	[99,143]
P45	32626	[99,143]
P45.2	33257	[143]
P46i(2)	33803	[101]
P46i(1)	34394	[101]
P46	35014	[99,143]
P47	36576	[99,143]
Pαgt	38430	[91]
PmobB (Pαgt.1)	38682	[99]
Pαgt.4	39447	[91]
P55	40180	[100]
P55.8(2)	42542	[101]
P55.8	42805	[100]
PnrdG (P55.9)	43023	[100]
PmobC	43744	[101]
PnrdD+ (P49.1)	6440	[144]
PnrdC(2)	48465	[101]
PnrdC(1)	48492	[101]
PnrdC.7	53325	[101]
PmobD	57389	[101]
PmobD.3	58381	[101]
Ptk.3	61076	[101]
Pvs.7	64382	[101]
PipIII	66724	[101]
PtRNAE (PtRNAsc1)	72593	[99]
P57A	74877	[99]
P1	75393	[99]
PrnlB	109763	[91]
P24.3	110108	[91]
Phoc	111757	[91]
PuvsY	115371	[8,99,126,145]
P30	127234	[102]
P30.2	128355	[102]
P31	131540	[146]
Pcd	132839	[91]
PI-TevIII (PnrdBin)	138939	[147]
PnrdB+	139878	[148]
PnrdA	142726	[100]
Ptd	145142	[100]
P32	148057*	[149]
PsegG	148678	[91]
PdsbA	149873	[129]
PdsbA(2)	149951	[91]
P34i	153011	[99]
P52	65227	[91]
Pndd.3	166702	[91]

Position of transcription start refers to T4 sequence [[4]; http://phage.bioc.tulane.edu/] Promoter names in parentheses refer to previous designations.

#### AsiA, the co-activator of T4 middle transcription

AsiA (*A*udrey *S*tevens *i*nhibitor or *a*nti-*s*igma *i*nhibitor) is a small protein of 90 residues. It was originally identified as a 10 kDa protein that binds very tightly to the σ^70 ^subunit of RNAP [11,58,59] with a ratio of 1:1 [60]. Later work indicated that a monomer of AsiA binds to C-terminal portions of σ^70^, Regions 4.1 and 4.2 [26,60-70]. In solution, AsiA is a homodimer whose self-interaction face is composed of mostly hydrophobic residues within the N-terminal half of the protein [65,71]. A similar face of AsiA interacts with σ^70 ^[26], suggesting that upon binding to σ^70^, a monomer of AsiA in the homodimer simply replaces its partner for σ^70^. Curiously, the AsiA structure also contains a helix-turn-helix motif (residues 30 to 59), suggesting the possibility of an interaction between AsiA and DNA [71]. However, as yet, no such interaction has been detected.

**Figure 3:**
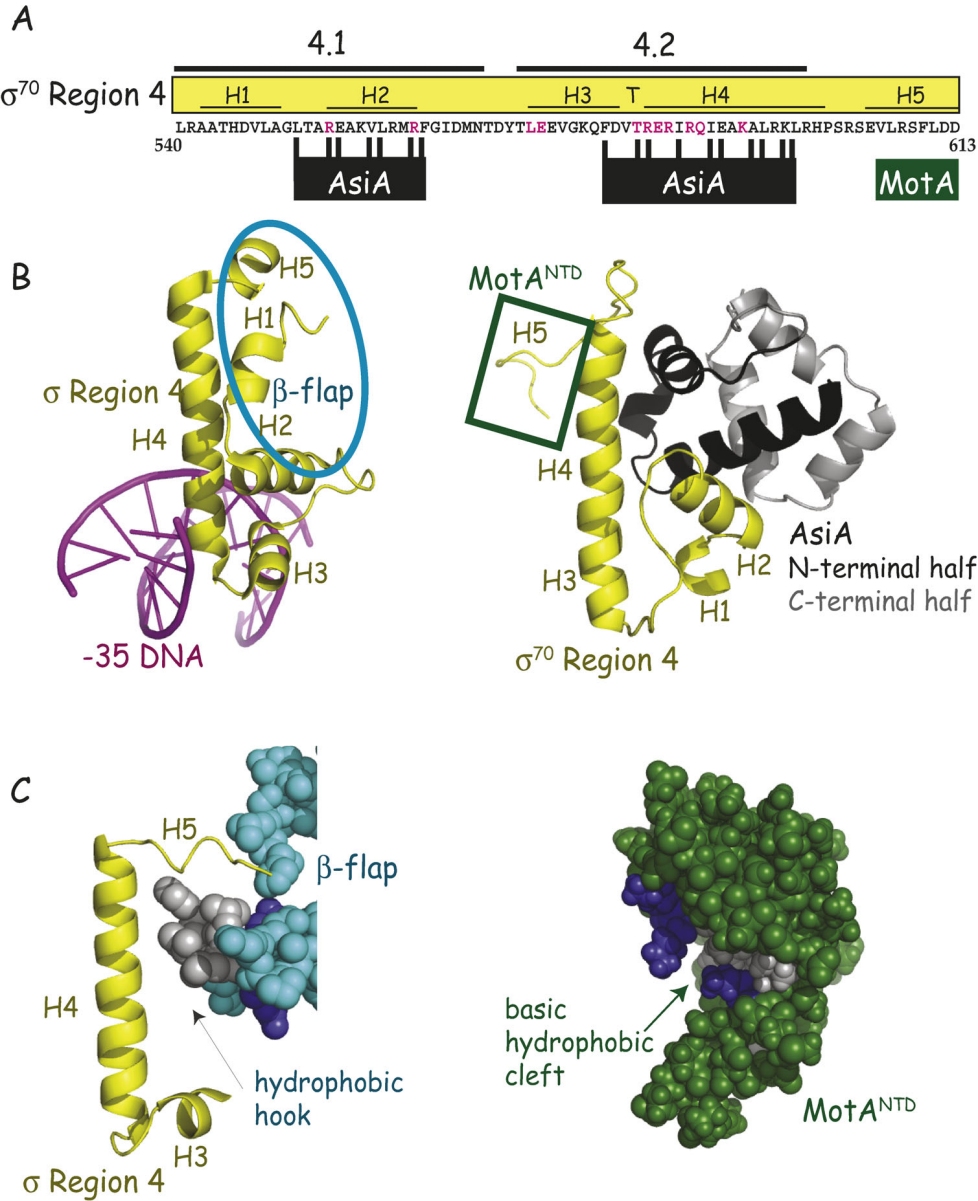
**Interaction of σ**^**70 **^**region 4 with -35 element DNA, the β-flap, AsiA and MotA**. A) Sequence of σ^70 ^Region 4 (residues 540-613) with subregions 4.1 and 4.2; the α helices H1 through H5 with a turn (T) between H3 and H4 are shown. Residues of σ^70 ^that interact with the -35 element [25] are colored in magenta. Residues that interact with AsiA [26] or the region that interacts with MotA [97,104] is indicated. B) Structures showing the interaction of *T. aquaticus *σ Region 4 with -35 element DNA [25] (left, accession # 1KU7) and interaction of σ^70 ^Region 4 with AsiA [26] (right, accession # 1TLH). σ, yellow; DNA, magenta; AsiA, N-terminal half in black, C-terminal half in gray. On the left, the portions of σ that interact with the β-flap (σ residues in and near H1, H2, and H5) are circled in turquoise; on the right, H5, the far C-terminal region of σ^70 ^that interacts with MotA, is in the green square. C) Structures showing the interaction of *T. thermophilus *σ H5 with the β-flap tip [22] (left, accession # 1IW7) and the structure of MotA^NTD ^[94] (right, accession # 1I1S) are shown. On the β-flap (left) and MotA^NTD ^(right) structures, hydrophobic residues (L, I, V, or F) and basic residues (K or R) are colored in gray or blue, respectively. The interaction site at the β-flap tip is a hydrophobic hook, while the structure in MotA^NTD ^is a hydrophobic cleft.

Multiple contacts make up the interaction between AsiA and σ^70 ^Region 4 (Figure 3A). The NMR structure (Figure 3B, right) reveals that 18 residues present in three α helices within the N-terminal half of AsiA (residues 10 to 42) contact 17 residues of σ^70 ^[26]. Biochemical analyses have confirmed that AsiA residues E10, V14, I17, L18, K20, F21, F36, and I40, which contact σ^70 ^Region 4 in the structure, are indeed important for the AsiA/σ^70 ^interaction and/or for AsiA transcriptional function *in vitro *[72-74]. Of all of these residues, I17 appears to be the most important, and thus, has been termed "the linchpin" of the AsiA/σ^70 ^Region 4 interaction [74]. A mutant AsiA missing the C-terminal 17 residues is as toxic as the full length protein when expressed *in vivo *[72,75], and even a mutant missing the C-terminal 44 residues is still able to interact with σ^70 ^Region 4 and to co-activate transcription weakly [72]. These results are consistent with the idea that only the N-terminal half of AsiA is absolutely required to form a functional AsiA/σ^70 ^complex. Together, the structural and biochemical work indicate that there is an extensive interface between the N-terminal half of AsiA and σ^70 ^Region 4, consistent with the early finding that AsiA copurifies with σ^70 ^until urea is added to dissociate the complex [76].

The σ^70 ^face of the AsiA/σ^70 ^complex includes residues in Regions 4.1 and 4.2 that normally contact the -35 DNA element or the β-flap of core [26] (Figure 3). Mutations within Region 4.1 or Region 4.2, which are at or near the AsiA contact sites in σ^70^, impair or eliminate AsiA function [77-79], providing biochemical evidence for these interactions. The structure of the AsiA/σ^70 ^Region 4 complex also reveals that AsiA binding dramatically changes the conformation of σ^70 ^Region 4, converting the DNA binding helix-turn-helix (Figure 3B, left) into one continuous helix (Figure 3B, right). Such a conformation would be unable to retain the typical σ^70 ^contacts with either the -35 DNA or with the β-flap. Thus, the association of AsiA with σ^70 ^should inhibit the binding of RNAP with promoters that depend on recognition of a -35 element. Indeed, early observations showed that AsiA functions as a transcriptional inhibitor at most promoters *in vitro *[9,10], blocking RPc formation [60], but TGn/-10 promoters, which are independent of a RNAP/-35 element contact, are immune to AsiA [62,66,80]. However, this result is dependent on the buffer conditions. In the presence of glutamate, a physiologically relevant anion that is known to facilitate protein-protein and protein-DNA interactions [81,82], extended incubations of AsiA-associated RNAP with -10/-35 and -35/TGn promoters eventually result in the formation of transcriptionally competent, open complexes that contain AsiA [72,83]. Under these conditions, AsiA inhibition works by significantly slowing the rate of RPo formation [83]. However, the formation of these complexes still relies on DNA recognition elements other than the -35 element (UP, TGn, and -10 elements), again demonstrating that AsiA specifically targets the interaction of RNAP with the -35 DNA.

Because AsiA strongly inhibits transcription from -35/-10 and -35/TGn promoters, expression of plasmid-encoded AsiA is highly toxic in *E. coli*. Thus, during infection, AsiA may serve to significantly inhibit host transcription. Although it might be reasonable to suppose that AsiA performs the same role at T4 early promoters, this is not the case. The shut-off of early transcription, which occurs a few minutes after infection, is still observed in a T4 *asiA*- infection [54], and early promoters are only modestly affected by AsiA *in vitro *[84]. This immunity to AsiA is probably due to the multiple RNAP recognition elements present in T4 early promoters (Figure 2). Thus, AsiA inhibition does not significantly contribute to the early to middle promoter transition. AsiA also does not help to facilitate the replacement of σ^70 ^by the T4-encoded late σ factor, which is needed for T4 late promoter activity [85], indicating that AsiA is not involved in the middle to late promoter transition.

Although AsiA was originally designated as an "anti-sigma" factor and is still frequently referred to as such, it is important to note that it behaves quite differently from classic anti-sigma factors. Unlike these factors, its binding to σ^70 ^does not prevent the σ^70^/core interaction; it does not sequester σ^70^. Instead it functions as a member of the RNAP holoenzyme. Consequently, AsiA is more correctly designated as a co-activator rather than an anti-sigma factor, and its primary role appears to be in activation rather than inhibition.

#### MotA, the transcriptional activator for middle promoters

The T4 *motA *(*m*odifier *o*f *t*ranscription) gene was first identified from a genetic selection developed to isolate mutations in T4 that increase the synthesis of the early gene product rIIA [86]. In fact, expression of several early genes increase in the T4 *motA- *infection, presumably because of a delay in the shift from early to middle transcription [87]. MotA is a basic protein of 211 amino acids, which is expressed as an early product [88]. The MotA mRNA is cleaved within its Shine-Dalgarno sequence by the T4 nuclease, RegB. Consequently, the burst of MotA protein synthesis, which occurs within the first couple minutes of infection [55], must be sufficient for all the subsequent MotA-dependent transcription.

MotA binds to a DNA recognition element, the MotA box, to activate transcription in the presence of AsiA-associated RNAP [7,8,11-13,89,90]. A MotA box consensus sequence of 5'(a/t)(a/t)(a/t)TGCTTtA3' [91] has been derived from 58 T4 middle promoters (Pm) (Table 1). This sequence is positioned 12 bp +/- 1 from the σ^70^-dependent -10 element,-12TAtaaT-7 (Figure 2). MotA functions as a monomer [92-94] with two distinct domains [95]. The N-terminal half of the protein, MotA^NTD ^contains the trans-activation function [96-98]. The structure of this region shows five α-helices, with helices 1, 3, 4, and 5 packing around the central helix 2 [93]. The C-terminal half, MotA^CTD^, binds MotA box DNA [97] and consists of a saddle-shaped, 'double wing' motif, three α-helices interspersed with six β-strands [94]. As information about MotA-dependent activation has emerged, it has become apparent that MotA differs from other activators of bacterial RNAP in several important aspects. The unique aspects of MotA are discussed below.

##### 1) MotA tolerates deviations within the MotA box consensus sequence

Early work [[3,99]; reviewed in [1]] identified a highly conserved MotA box sequence of (a/t)(a/t)TGCTT(t/c)a with an invariant center CTT based on more than twenty T4 middle promoters. However, subsequent mutational analyses revealed that most single bp changes within the consensus sequence, even within the center CTT, are well-tolerated for MotA binding and activation *in vitro *[100]. Furthermore, several active middle promoters have been identified whose MotA boxes deviate significantly from the consensus, confirming that MotA is indeed tolerant of bp changes *in vivo *[91,100-102].

An examination of the recognized base determinants within the MotA box has revealed that MotA senses minor groove moieties at positions -32 and -33 and major groove determinants at positions -28 and -29 [103]. (For this work, the MotA box was located at positions -35 to -26, its position when it is present 13 bp upstream of the -10 element.) In particular, the 5-Me on -29 T contributes to MotA binding. However, despite its high conservation, there seems to be little base recognition of -31 G:C, -30 C:G at the center of the MotA box. In wt T4 DNA, each cytosine in this sequence is modified by the presence of a hydroxymethylated, glucosylated moiety at cytosine position 5. This modification places a large, bulky group within the major groove, making it highly unlikely that MotA could contact a major groove base determinant at these positions. In addition, MotA binds and activates transcription using unmodified DNA; thus, the modification itself cannot be required for function. However, for two specific sequences, DNA modification does seem to affect MotA activity. One case is the middle promoter upstream of gene 46, P46. The MotA box within P46 contains the unusual center sequence ACTT rather than the consensus GCTT. MotA binds a MotA box with the ACTT sequence poorly, and MotA activation of P46 *in vitro *using wt T4 DNA is significantly better than that observed with unmodified DNA [100]. These results suggest that DNA modification may be needed for full activity of the ACTT MotA box motif. On the other hand, when using unmodified DNA *in vitro*, MotA binds a MotA box with a center sequence of GATT nearly as well as one with the consensus GCTT sequence, and a promoter with the GATT motif is fully activated by MotA *in vitro*. However, several potential T4 middle promoter sequences with a GATT MotA box and an excellent σ^70^-dependent -10 element are present within the T4 genome, but these promoters are not active [100]. This result suggests that the cytosine modification opposite the G somehow "silences" GATT middle promoter sequences.

##### 2) MotA is not a strong DNA-binding protein

In contrast to many other well-characterized activators of *E. coli *RNAP, MotA has a high apparent dissociation constant for its binding site (100 - 600 nM [92,103,104]), and a large excess of MotA relative to DNA is needed to detect a MotA/DNA complex in a gel retardation assay or to detect protein protection of the DNA in footprinting assays [90]. In contrast, stoichiometric levels of MotA are sufficient for transcription *in vitro *[90]. These results are inconsistent with the idea that the tight binding of MotA to a middle promoter recruits AsiA-associated RNAP for transcription. In fact, in nuclease protection assays, MotA binding to the MotA box of a middle promoter is much stronger in the presence of AsiA and RNAP than with MotA alone [89,90]. Furthermore, in contrast to the sequence deviations permitted within the MotA box, nearly all middle promoters have a stringent requirement for an excellent match to the σ^70^-dependent -10 element [91,100,101]. This observation suggests that the interaction of σ^70 ^Region 2.4 with its cognate -10 sequence contributes at least as much as MotA binding to the MotA box in the establishment of a stable RNAP/MotA/AsiA/Pm complex.

##### 3) The MotA binding site on σ^70 ^is unique among previously characterized activators of RNAP

Like many other characterized activators, MotA interacts with σ^70 ^residues within Region 4 to activate transcription. However, other activators target basic σ^70 ^residues from 593 to 603 within Region 4.2 that are immediately C-terminal to residues that interact specifically with the -35 element DNA [27,105-112] [Figure 3A; reviewed in [113]]. In contrast, the interaction site for MotA is a hydrophobic/acidic helix (H5) located at the far C-terminus of σ^70 ^(Figure 3A). MotA^NTD ^interacts with this region *in vitro *and mutations within σ^70 ^H5 impair both MotA binding to σ^70 ^and MotA-dependent transcription [77,97,104]. In addition, a mutation within H5 restores infectivity of a T4 *motA- *phage in a particular strain of *E. coli*, TabG [114], which does not support T4 *motA- *growth [115].

Recent structural and biochemical work has indicated that a basic/hydrophobic cleft within MotA^NTD ^contains the molecular face that interacts with σ^70 ^H5 (Figure 3C, right). Mutation of MotA residues K3, K28, or Q76, which lie in this cleft, impair the ability of MotA to interact with σ^70 ^H5 and to activate transcription, and render the protein incapable of complementing a T4 *motA- *phage for growth [104]. Interestingly, substitutions of MotA residues D30, F31, and D67, which lie on another exposed surface outside of this cleft, also have deleterious effects on the interaction with σ^70^, transcription, and/or phage viability [98,104]. These residues are contained within a hydrophobic, acidic patch, which may also be involved in MotA activation or another unidentified function of MotA.

#### The process of sigma appropriation

The mechanism of MotA-dependent activation occurs through a novel process, called sigma appropriation [reviewed in [2]]. Insight into this process began with the finding that some middle promoters function *in vitro *with RNAP alone. The middle promoter P_uvsX_, which is positioned upstream of the T4 recombination gene *uvsX*, is such a promoter [13]. This promoter is active because it has UP elements and a perfect -10 element to compensate for its weak homology to a σ^70 ^-35 sequence. (It should be noted that significant activity of P_uvsX _and other middle promoters in the absence of MotA/AsiA is only seen when using unmodified DNA, because the modification present in T4 DNA obscures needed major grove contacts for RNAP.) Using unmodified P_uvsX _DNA, it has been possible to investigate how the presence of MotA and AsiA alone and together affect the interactions between RNAP and a middle promoter [72,89,90,103]. The RPo formed by RNAP and P_uvsX _exhibits protein/DNA contacts that are similar to those seen using a typical -35/-10 promoter; addition of MotA in the absence of AsiA does not significantly alter these contacts. As expected, addition of AsiA without MotA inhibits the formation of a stable complex. However, in the presence of both MotA and AsiA, a unique RPo is observed. This MotA/AsiA activated complex has the expected interactions between RNAP and the -10 element, but it has unique protein-DNA interactions upstream of the -10 element. In particular, σ^70 ^Region 4 does not make its usual contacts with the -35 element DNA; rather MotA binds to the MotA box that overlaps the -35 sequence. As expected, when using fully ADP-ribosylated RNAP there is an abrupt loss of footprint protection just upstream of the MotA box in P_uvsX_, consistent with the loss of UP element interactions when both α-CTD's are modified; when using RNAP that has not been ADP-ribosylated, the UP elements in P_uvsX _are protected.

**Figure 4:**
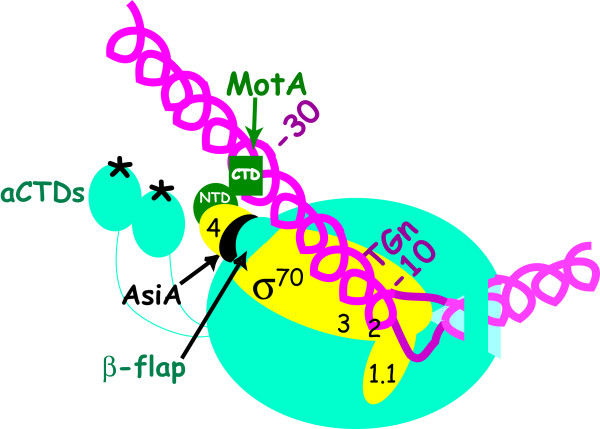
**σ appropriation at a T4 middle promoter**. Cartoon depicting a model of RPo at a T4 middle promoter (colors as in Fig. 1). Interaction of AsiA with σ^70 ^Region 4 remodels Region 4, preventing its interaction with the β-flap or with the -35 region of the DNA. This interaction then facilitates the interaction of MotA^NTD ^with σ^70 ^H5 and MotA^CTD ^with the MotA box centered at -30. Protein-DNA interactions at σ^70 ^promoter elements downstream of the MotA box (the TGn and -10 elements) are not significantly affected. ADP-ribosylation of Arg265 on each α-CTD, catalyzed by the T4 Alt and ModA proteins, is denoted by the asterisks. The modification prevents the α subunits from interacting with DNA upstream of the MotA box.

Taken together, these biochemical studies argued that within the activated complex, σ^70 ^Region 2.4 binds tightly to the σ^70^-dependent -10 element, but the MotA/MotA box interaction is somehow able to replace the contact that is normally made between σ^70 ^Region 4 and the -35 DNA (Figure 4) [89,103]. The subsequent AsiA/σ^70 ^Region 4 structure [26] (Figure 3B, right) shows just how this can be done. Through its multiple contacts with σ^70 ^residues in Regions 4.1 and 4.2, AsiA remodels Region 4 of σ^70^. When the AsiA/σ^70 ^complex then binds to core, σ^70 ^Region 4 is incapable of forming its normal contacts with -35 element DNA (Figure 3B, left). In addition, the restructuring of σ^70 ^Region 4 prevents its interaction with the β-flap, allowing the far C-terminal region H5 of σ^70 ^to remain available for its interaction with MotA. Consequently, in the presence of AsiA-associated RNAP, MotA can interact both with the MotA box and with σ^70 ^H5 [77,97,104].

Recent work has suggested that additional portions of AsiA, MotA and RNAP may be important for σ appropriation. First, the C-terminal region of AsiA (residues 74-90) may contribute to activation at P_uvsX _by directly interacting both with the β-flap and with MotA^NTD^. In particular, the AsiA N74D substitution reduces an AsiA/β-flap interaction observed in a 2-hybrid assay and impairs the ability of AsiA to inhibit transcription from a -35/-10 promoter *in vitro *[116]. This mutation also renders AsiA defective in co-activating transcription from P_uvsX _*in vitro *if it is coupled with a σ^70 ^F563Y substitution that weakens the interaction of AsiA with σ^70 ^Region 4 [117]. On the other hand, an AsiA protein with either a M86T or R82E substitution has a reduced capacity to interact with MotA^NTD ^in a 2-hybrid assay and yields reduced levels of MotA/AsiA activated transcription from P_uvsX _*in vitro *[118]. The M86 and R82 mutations do not affect the interaction of AsiA with σ^70 ^or with the β-flap, and they do not compromise the ability of AsiA to inhibit transcription [118], suggesting that they specifically affect the interaction with MotA. These results argue that AsiA serves as a bridge, which connects σ^70^, the β-flap, and MotA. However, in other experiments, MotA/AsiA activation of P_uvsX _is not affected when using AsiA proteins with deletions of this C-terminal region (Δ79-90 and Δ74-90), and even AsiA Δ47-90 still retains some ability to co-activate transcription [72]. Furthermore, the C-terminal half of the AsiA ortholog of the *vibrio *phage KVP40 (discussed below) has little or no sequence homology with its T4 counterpart yet in the presence of T4 MotA and *E. coli *RNAP, it effectively co-activates transcription from P_uvsX _*in vitro *[119], and NMR analyses indicate that the addition of MotA to the AsiA/σ^70 ^Region 4 complex does not significantly perturb chemical shifts of AsiA residues [104]. Thus, further work is needed to clarify the role of the of AsiA C-terminal region. Finally, very recent work has shown that the inability of T4 *motA *mutants to plate on the TabG strain arises from a G1249D substitution within β, thereby implicating a region of β that is distinct from the β-flap in MotA/AsiA activation [120]. This mutation is located immediately adjacent to a hydrophobic pocket, called the Switch 3 loop, which is thought to aid in the separation of the RNA from the DNA-RNA hybrid as RNA enters the RNA exit channel [28]. The presence of the β G1249D mutation specifically impairs transcription from T4 middle promoters *in vivo*, but whether the substitution directly or indirectly affects protein-protein interactions is not yet known [120]. Taken together, these results suggest that MotA/AsiA activation employs multiple contacts, some of which are essential under all circumstances (AsiA with σ^70 ^Regions 4.1 and 4.2, MotA with σ^70 ^H5) and some of which may provide additional contacts perhaps under certain circumstances to strengthen the complex.

Concurrent work with the T4 middle promoter P_rIIB2 _has yielded somewhat different findings than those observed with P_uvsX _[121]. P_rIIB2 _is a TGn/-10 promoter that does not require an interaction between σ^70 ^Region 4 and the -35 element for activity. Thus, the presence of AsiA does not inhibit RPo formation at this promoter. An investigation of the complexes formed at P_rIIB2 _using surface plasmon resonance revealed that MotA and AsiA together stimulate the initial recognition of the promoter by RNAP. In addition, *in vitro *transcription experiments indicated that MotA and AsiA together aid in promoter clearance, promoting the formation of the elongating complex. Thus, MotA may activate different steps in initiation, depending on the type of promoter. However, there is no evidence to suggest that the protein/protein and protein/DNA contacts are significantly different with different middle promoters.

Interestingly, AsiA binds rapidly to σ^70 ^when σ^70 ^is free, but binds poorly, if at all, to σ^70 ^that is present in RNAP [122]. The inability of AsiA to bind to σ^70 ^within holoenzyme may be useful for the phage because it ties the activation of middle promoters to the efficiency of early transcription. This stems from the fact that σ^70 ^is usually released from holoenzyme once RNAP has cleared a promoter [[123] and references therein]. Since there is an excess of core relative to σ factors, there is only a brief moment for AsiA to capture σ^70^. Consequently, the more efficiently the T4 early promoters fire, the more opportunities are created for AsiA to bind to σ^70^, which then leads to increased MotA/AsiA-dependent middle promoter transcription.

#### Sigma appropriation in other T4-type phages

Although hundreds of activators of bacterial RNAP are known, the T4 MotA/AsiA system represents the first identified case of sigma appropriation. A search for MotA and AsiA orthologs has revealed several other T4-type phage genomes that contain both *motA *and *asiA *genes [[124] and http://phage.bioc.tulane.edu/]. These range from other coliphages (RB51, RB32, and RB69) to more distantly related phages that infect *aeromonas *(PHG25, PHG31, and 44RR) and *acinetobacter *(PHG133). In addition, orthologs for *asiA *have also been found in the genomes of the *vibrio *phages KVP40 and NT1 and the *aeromonas *phages PHG65 and Aeh1, even though these genomes do not have a recognizable *motA*. The KVP40 AsiA protein shares only 27% identity with its T4 counterpart. However, it inhibits transcription by *E. coli *RNAP alone and co-activates transcription with T4 MotA as effectively as T4 AsiA [119]. Thus, it may be that KVP40 and other phages that lack a MotA sequence homolog, do in fact have a functional analog of the MotA protein. Alternatively, the KVP40 AsiA may serve only as an inhibitor of transcription.

No examples of sigma appropriation outside of T4-type phage have been discovered. Although sequence alignments suggested that the *E. coli *anti-sigma protein Rsd, which also interacts with σ^70^, may be a distant member of the AsiA family [119], a structure of the Rsd/sigma Region 4 complex is not consistent with this idea [30]. Recent work has identified a protein (CT663) involved in the developmental pathway of the human pathogen *Chlamydia trachomatis *that shares functional features with AsiA [125]. It binds both to Region 4 of the primary σ (σ^66^) of *C. trachomatis *and to the β-flap of core, and it inhibits σ^66^-dependent transcription. More importantly, like AsiA, it works by remaining bound to the RNAP holoenzyme rather than by sequestering σ^66^.

### Transcription of middle genes by the extension of early transcripts

Even though the expression of middle genes is highly dependent on the activation of middle promoters, isolated mutations within *motA *and *asiA *are surprisingly not lethal. Such mutant phage show a DNA delay phenotype, producing tiny plaques on wt *E. coli *[11,87]. The replication defect reflects the reduced level of T4 replication proteins, whose genes have MotA-dependent middle promoters. In addition, two T4 replication origins are driven by MotA-dependent transcription from the middle promoters, P_uvsY _and P_34i _[126]. However, deletion of either *motA *[127] or *asiA *[54] is lethal. Recent work suggests that leakiness of the other nonsense and temperature sensitive mutations provide enough protein for minimal growth [120].

Besides MotA-dependent promoters, middle RNA is also generated by the extension of early transcripts into middle genes. This is because most, if not all, middle genes are positioned downstream of early gene(s) and early promoters. Production of this extended RNA is time-delayed relative to the RNA from the upstream "immediate early (IE)" gene. Thus, middle RNA generated from this extension was originally designated "delayed early" (DE), since it cannot be synthesized until the elongating RNAP reaches the downstream gene(s). Early work (reviewed in [1]) classified genes as IE, DE, or middle based on when and under what conditions the RNA or the encoded protein was observed. IE RNA represents transcripts that are detected immediately after infection and do not require phage protein synthesis. DE RNA requires phage protein synthesis, but this RNA and DE gene products are still detected in a T4 *motA- *infection. In contrast, the expression of genes that were classified as "middle" is significantly reduced in a T4 motA- infection. In addition, while both DE and "middle" RNA arise after IE transcription, the peak of the RNA that is substantially dependent on MotA is slightly later and lasts somewhat longer than the DE peak. However, it should be noted that these original designations of genes as DE or middle are now known to be somewhat arbitrary. Many, if not all, of these genes are transcribed from both early and middle promoters. In fact, while a microarray analysis investigating the timing of various prereplicative RNAs [128] was generally consistent with known Pe and Pm promoters [4], there were a number of discrepancies, especially between genes that were originally classified as either "DE" or "middle". Thus, it is now clear that both the extension of early transcripts and the activation of middle promoters is important for the correct level of middle transcription.

Early experiments [summarized in [1]] offered evidence that DE RNA synthesis might require a T4 system to overcome Rho-dependent termination sites located between IE and DE genes. First, the addition of chloramphenicol at the start of a T4 infection prevents the generation of DE RNAs, indicating a requirement for protein synthesis and suggesting that phage-encoded factor(s) might be needed for the extension of IE RNAs. Second, in a purified *in vitro *system using RNAP and T4 DNA, both IE and DE RNA are synthesized unless the termination factor Rho is added. Addition of Rho restricts transcription to IE RNA, indicating that Rho-dependent termination sites are located upstream of DE genes. Third, DE RNA from a specific promoter upstream of gene 32 is not observed in a T4 *motA- *infection, suggesting that MotA itself may be needed to form or stabilize this DE RNA [129]. It is unlikely that a MotA-dependent gene product, rather than MotA, is responsible for this effect, since the DE transcripts are synthesized before or simultaneously with the activation of middle promoters. Finally, wt T4 does not grow in particular *rho *mutant alleles, called *nusD*, that produce Rho proteins with altered activity, and the level of certain DE RNAs and DE gene products in T4/*nusD *infections is depressed. An initial interpretation of this result was that there is more Rho-dependent termination in a *nusD *allele, which then depresses the level of DE RNA. T4 suppressors that grow in *nusD *were subsequently isolated and found to contain mutations within the T4 *comC-α *(also called *goF*) gene [130,131], which expresses an early product.

Given all of these findings, it was postulated that T4 uses an anti-termination system, perhaps like the N or Q systems of phage λ [reviewed in [132]], to actively prevent Rho-dependent termination and that MotA, ComC-α, or another protein is involved in this process. However, *comC-α *is not essential, and the addition of amino acid analogs, which would generate nonfunctional proteins, has been shown to be sufficient for the synthesis of at least certain DE RNAs [reviewed in [1]]. These results suggest that at least in some cases, translation is simply needed to prevent polarity; consequently, the process of translation itself, rather than a specific factor(s), is sufficient to inhibit Rho termination. If so, the loss of DE RNA observed in the presence of Rho *in vitro *would be due to the lack of coupled transcription/translation. Thus, when the upstream gene is being translated in an infection *in vivo*, Rho RNA binding sites would be occluded by ribosomes and consequently unavailable.

More recent work has suggested that Rho may affect DE RNA *in vivo *because of its ability to bind RNA rather than its termination activity [133,134]. Sequencing of the *rho *gene in six *nusD *alleles has revealed that in five cases, the *rho *mutation lies within the RNA-binding site of Rho. Furthermore, the addition of such a mutant Rho protein to an *in vitro *transcription system does not produce more termination but rather results in an altered and complicated pattern of termination. There is actually less termination at legitimate Rho-dependent termination sites, but in some cases, more termination at other sites. Unexpectedly, increasing the amount of the mutant Rho proteins rescues T4 growth in a *nusD *allele, a result that is not compatible with the mutant Rho promoting more termination. In addition, expression of the Rop protein, an RNA-binding protein encoded by the pBR322 plasmid, also rescues T4 growth in *nusD*.

Taken together, these results have led to another hypothesis to explain DE RNA. In this model, T4 DE transcripts *in vivo *are susceptible to nuclease digestion and require a process to limit this degradation. Active translation can prevent this nuclease attack, thus explaining the loss of DE RNA in the presence of chloramphenicol. In addition, a protein that can bind RNA, such as wt Rho, Rop, or perhaps the mutated T4 ComC-α, may also be useful. Thus, the *nusD *Rho proteins are defective not because they terminate IE transcripts more effectively, but because they have lost the ability of wt Rho to bind and somehow protect the RNA. However, it should be noted that as of yet, there is no evidence identifying a particular nuclease(s) involved in this model. Furthermore, the function of wt *comC-α *or exactly how Rho or Rop "protect" DE RNA is not known. Recent work has shown that both transcription termination and increased mRNA stability by RNA-binding proteins are involved in the regulation of gene expression in eukaryotes and their viruses [135,136]. A thorough investigation of these processes in the simple T4 system could provide a powerful tool to understanding this mode of gene regulation.

## Conclusion

T4 regulates its development and the timed expression of prereplicative genes by a sophisticated process. In the past few years, we have learned how T4 employs several elegant strategies, from encoding factors to alter the host RNAP specificity to simply degrading the host DNA, in order to overtake the host transcriptional machinery. Some of these strategies have revealed unexpected and fundamentally significant findings about RNAP. For example, studies with T4 early promoters have challenged previous ideas about how the α-CTDs of RNAP affect transcription. Work with host promoters argued that contact between the α-CTDs of RNAP and promoter UP elements or certain activators increases transcription; in particular, α residue Arg265 was crucial for this interaction. Thus, one would expect that modification of Arg265 would depress transcription. However, the activity of certain T4 early promoters actually increases when Arg265 of one of the two RNAP α subunits is ADP-ribosylated. This finding underscores our limited understanding of α-CTD function and highlights how T4 can provide a tool for investigating this subunit of RNAP.

The T4 system has also revealed a previously unknown method of transcription activation called sigma appropriation. This process is characterized by the binding of a small protein, T4 AsiA, to Region 4 of the σ^70 ^subunit of RNAP, which then remodels this portion of polymerase. The conformation of Region 4 in the AsiA/σ^70 ^Region 4 structure differs dramatically from that seen in other structures of primary σ factors and demonstrates that Region 4 has a previously unknown flexibility. Furthermore, studies with the T4 MotA activator have identified the far C-terminal region of σ^70 ^as a target for activation. Prior to the T4 work, it was thought that this portion of σ^70^, which is normally embedded within the β-flap "hook" of core, is unavailable. Based on the novel strategy T4 employs to activate its middle promoters, we now know how a domain within RNAP can be remodeled and then exploited to alter promoter specificity. It may be that other examples of this type of RNAP restructuring will be uncovered.

The core subunits of bacterial RNAP are generally conserved throughout biology both in structure and in function [reviewed in [137,138]]. In addition, it is now apparent that eukaryotic RNAP II employs protein complexes that function much like σ factors to recognize different core promoter sequences [[139,140] and references therein]. Thus, the T4 system, which is simple in components yet complex in details, provides an amenable resource for answering basic questions about the complicated process of transcriptional regulation. Using this system, we have been able to uncover at a molecular level many of the protein/protein and protein/DNA interactions that are needed to convert the host RNAP into a RNAP that is dedicated to the phage. This work has given us "snapshots" of the transcriptionally competent protein/DNA complexes generated by the actions of the T4 proteins. The challenge in the future will be to understand at a detailed mechanistic level how these interactions modulate the various "nuts and bolts" of the RNAP machine.

## List of abbreviations

bp: base pair(s); ds: double-stranded; ss: single-stranded; RPo: open complex; RPc: closed complex; R or RNAP: RNA polymerase; P: promoter; TGn: -15TGn-13 (extended -10 motif); Pe: T4 early promoter; Pm: T4 middle promoter; rNTPs: ribonucleoside triphosphates; wt: wild type.

## Competing interests

The author declares that they have no competing interests.

## Authors' contributions

DH is solely responsible for this manuscript.

## References

[B1] Stitt B Hinton DM Karam JD, Drake J, Kreuzer KN, Mosig G, Hall D, Eiserling F, Black L, Spicer E, Kutter E, Carlson K, Miller ES Regulation of middle-mode transcription Molecular biology of bacteriophage T4 1994 Washington, D.C.: American Society for Microbiology 142 160

[B2] Hinton DM Pande S Wais N Johnson XB Vuthoori M Makela A Hook-Barnard I Transcriptional takeover by sigma appropriation: remodelling of the sigma70 subunit of Escherichia coli RNA polymerase by the bacteriophage T4 activator MotA and co-activator AsiA Microbiology 2005 151 1729 1740 10.1099/mic.0.27972-0 15941982

[B3] Brody E Rabussay D Hall D Mathews CK, Kutter EM, Mosig G, Berget PB Regulation of transcription of prereplicative genes Bacteriophage T4 1983 Washington, D. C.: American Society for Microbiology 174 183

[B4] Miller ES Kutter E Mosig G Arisaka F Kunisawa T Ruger W Bacteriophage T4 genome Microbiol Mol Biol Rev 2003 67 86 156 10.1128/MMBR.67.1.86-156.2003 12626685 PMC150520

[B5] Wilkens K Ruger W Karam JD, Drake JW, Kreuzer KN, Mosig G, Hall DH, Eiserling FA, Black LW, Spicer EK, Kutter E, Carlson K, Miller ES Transcription from early promoters Molecular Biology of Bacteriophage T4 1994 Washington, D. C.: American Society for Microbiology 132 141

[B6] Weisberg R Hinton DM Adhya S Mahy BWJ, van Regenmortel MHV Transcriptional Regulation in Bacteriophage Encyclopedia of Virology 2008 3 Oxford: Elsevier 174 186 174-186full_text

[B7] Hinton DM Transcription from a bacteriophage T4 middle promoter using T4 motA protein and phage-modified RNA polymerase J Biol Chem 1991 266 18034 18044 1917941

[B8] Schmidt RP Kreuzer KN Purified MotA protein binds the -30 region of a bacteriophage T4 middle-mode promoter and activates transcription in vitro J Biol Chem 1992 267 11399 11407 1597469

[B9] Stevens A New small polypeptides associated with DNA-dependent RNA polymerase of Escherichia coli after infection with bacteriophage T4 Proc Natl Acad Sci USA 1972 69 603 607 10.1073/pnas.69.3.603 4551978 PMC426516

[B10] Stevens A An inhibitor of host sigma-stimulated core enzyme activity that purifies with DNA-dependent RNA polymerase of E. coli following T4 phage infection Biochem Biophys Res Commun 1973 54 488 493 10.1016/0006-291X(73)91447-2 4585685

[B11] Ouhammouch M Orsini G Brody EN The asiA gene product of bacteriophage T4 is required for middle mode RNA synthesis J Bacteriol 1994 176 3956 3965 8021178 10.1128/jb.176.13.3956-3965.1994 PMC205593

[B12] Ouhammouch M Adelman K Harvey SR Orsini G Brody EN Bacteriophage T4 MotA and AsiA proteins suffice to direct Escherichia coli RNA polymerase to initiate transcription at T4 middle promoters Proc Natl Acad Sci USA 1995 92 1451 1455 10.1073/pnas.92.5.1451 7877999 PMC42537

[B13] Hinton DM March-Amegadzie R Gerber JS Sharma M Bacteriophage T4 middle transcription system: T4-modified RNA polymerase; AsiA, a sigma 70 binding protein; and transcriptional activator MotA Methods Enzymol 1996 274 43 57 full_text 8902795 10.1016/s0076-6879(96)74007-7

[B14] Browning DF Busby SJ The regulation of bacterial transcription initiation Nat Rev Microbiol 2004 2 57 65 10.1038/nrmicro787 15035009

[B15] Murakami KS Darst SA Bacterial RNA polymerases: the wholo story Curr Opin Struct Biol 2003 13 31 39 10.1016/S0959-440X(02)00005-2 12581657

[B16] Young BA Gruber TM Gross CA Views of transcription initiation Cell 2002 109 417 420 10.1016/S0092-8674(02)00752-3 12086598

[B17] Paget MS Helmann JD The sigma70 family of sigma factors Genome Biol 2003 4 203 10.1186/gb-2003-4-1-203 12540296 PMC151288

[B18] Gruber TM Gross CA Multiple sigma subunits and the partitioning of bacterial transcription space Annu Rev Microbiol 2003 57 441 466 10.1146/annurev.micro.57.030502.090913 14527287

[B19] Campbell EA Westblade LF Darst SA Regulation of bacterial RNA polymerase sigma factor activity: a structural perspective Curr Opin Microbiol 2008 11 121 127 10.1016/j.mib.2008.02.016 18375176 PMC2386898

[B20] Hook-Barnard IG Hinton DM Transcription Initiation by Mix and Match Elements: Flexibility for Polymerase Binding to Bacterial Promoters Gene Regulation and Systems Biology 2007 http://la-press.com/article.php?article_id=481:275-293 19119427 PMC2613000

[B21] Helmann JD RNA polymerase: a nexus of gene regulation Methods 2009 47 1 5 10.1016/j.ymeth.2008.12.001 19070783 PMC3022018

[B22] Vassylyev DG Sekine S Laptenko O Lee J Vassylyeva MN Borukhov S Yokoyama S Crystal structure of a bacterial RNA polymerase holoenzyme at 2.6 A resolution Nature 2002 417 712 719 10.1038/nature752 12000971

[B23] Murakami KS Masuda S Campbell EA Muzzin O Darst SA Structural basis of transcription initiation: an RNA polymerase holoenzyme-DNA complex Science 2002 296 1285 1290 10.1126/science.1069595 12016307

[B24] Murakami KS Masuda S Darst SA Structural basis of transcription initiation: RNA polymerase holoenzyme at 4 A resolution Science 2002 296 1280 1284 10.1126/science.1069594 12016306

[B25] Campbell EA Muzzin O Chlenov M Sun JL Olson CA Weinman O Trester-Zedlitz ML Darst SA Structure of the bacterial RNA polymerase promoter specificity sigma subunit Mol Cell 2002 9 527 539 10.1016/S1097-2765(02)00470-7 11931761

[B26] Lambert LJ Wei Y Schirf V Demeler B Werner MH T4 AsiA blocks DNA recognition by remodeling sigma(70) region 4 Embo J 2004 23 2952 2962 10.1038/sj.emboj.7600312 15257291 PMC514929

[B27] Jain D Nickels BE Sun L Hochschild A Darst SA Structure of a ternary transcription activation complex Mol Cell 2004 13 45 53 10.1016/S1097-2765(03)00483-0 14731393

[B28] Vassylyev DG Vassylyeva MN Perederina A Tahirov TH Artsimovitch I Structural basis for transcription elongation by bacterial RNA polymerase Nature 2007 448 157 162 10.1038/nature05932 17581590

[B29] Malhotra A Severinova E Darst SA Crystal structure of a sigma 70 subunit fragment from E. coli RNA polymerase Cell 1996 87 127 136 10.1016/S0092-8674(00)81329-X 8858155

[B30] Patikoglou GA Westblade LF Campbell EA Lamour V Lane WJ Darst SA Crystal Structure of the Escherichia coli Regulator of sigma(70), Rsd, in Complex with sigma(70) Domain 4 J Mol Biol 2007 372 649 659 10.1016/j.jmb.2007.06.081 17681541 PMC2083641

[B31] Sasse-Dwight S Gralla JD KMnO4 as a probe for lac promoter DNA melting and mechanism in vivo J Biol Chem 1989 264 8074 8081 2722774

[B32] Kontur WS Saecker RM Davis CA Capp MW Record MT Jr Solute probes of conformational changes in open complex (RPo) formation by Escherichia coli RNA polymerase at the lambdaPR promoter: evidence for unmasking of the active site in the isomerization step and for large-scale coupled folding in the subsequent conversion to RPo Biochemistry 2006 45 2161 2177 10.1021/bi051835v 16475805 PMC2631401

[B33] Saecker RM Tsodikov OV McQuade KL Schlax PE Jr Capp MW Record MT Jr Kinetic studies and structural models of the association of E. coli sigma(70) RNA polymerase with the lambdaP(R) promoter: large scale conformational changes in forming the kinetically significant intermediates J Mol Biol 2002 319 649 671 10.1016/S0022-2836(02)00293-0 12054861

[B34] Erie DA The many conformational states of RNA polymerase elongation complexes and their roles in the regulation of transcription Biochim Biophys Acta 2002 1577 224 239 12213654 10.1016/s0167-4781(02)00454-2

[B35] Landick R The regulatory roles and mechanism of transcriptional pausing Biochem Soc Trans 2006 34 1062 1066 10.1042/BST0341062 17073751

[B36] Ciampi MS Rho-dependent terminators and transcription termination Microbiology 2006 152 2515 2528 10.1099/mic.0.28982-0 16946247

[B37] Gilmour DS Fan R Derailing the locomotive: transcription termination J Biol Chem 2008 283 661 664 10.1074/jbc.R700032200 17998201

[B38] Adhya S Gottesman M Control of transcription termination Annu Rev Biochem 1978 47 967 996 10.1146/annurev.bi.47.070178.004535 354508

[B39] Wilkens K Ruger W Characterization of bacteriophage T4 early promoters in vivo with a new promoter probe vector Plasmid 1996 35 108 120 10.1006/plas.1996.0013 8700964

[B40] Koch T Raudonikiene A Wilkens K Ruger W Overexpression, purification, and characterization of the ADP-ribosyltransferase (gpAlt) of bacteriophage T4: ADP-ribosylation of E. coli RNA polymerase modulates T4 "early" transcription Gene Expr 1995 4 253 264 7787417 PMC6134386

[B41] Horvitz HR Bacteriophage T4 mutants deficient in alteration and modification of the Escherichia coli RNA polymerase J Mol Biol 1974 90 739 750 10.1016/0022-2836(74)90537-3 4615179

[B42] Horvitz HR Control by bacteriophage T4 of two sequential phosphorylations of the alpha subunit of Escherichia coli RNA polymerase J Mol Biol 1974 90 727 738 10.1016/0022-2836(74)90536-1 4615178

[B43] Depping R Lohaus C Meyer HE Ruger W The mono-ADP-ribosyltransferases Alt and ModB of bacteriophage T4: target proteins identified Biochem Biophys Res Commun 2005 335 1217 1223 10.1016/j.bbrc.2005.08.023 16112649

[B44] Ross W Gosink KK Salomon J Igarashi K Zou C Ishihama A Severinov K Gourse RL A third recognition element in bacterial promoters: DNA binding by the alpha subunit of RNA polymerase Science 1993 262 1407 1413 10.1126/science.8248780 8248780

[B45] Gaal T Ross W Blatter EE Tang H Jia X Krishnan VV Assa-Munt N Ebright RH Gourse RL DNA-binding determinants of the alpha subunit of RNA polymerase: novel DNA-binding domain architecture Genes Dev 1996 10 16 26 10.1101/gad.10.1.16 8557191

[B46] Murakami K Fujita N Ishihama A Transcription factor recognition surface on the RNA polymerase alpha subunit is involved in contact with the DNA enhancer element EMBO J 1996 15 4358 4367 8861963 PMC452160

[B47] Zou C Fujita N Igarashi K Ishihama A Mapping the cAMP receptor protein contact site on the alpha subunit of Escherichia coli RNA polymerase Mol Microbiol 1992 6 2599 2605 10.1111/j.1365-2958.1992.tb01437.x 1333035

[B48] Tiemann B Depping R Gineikiene E Kaliniene L Nivinskas R Ruger W ModA and ModB, two ADP-ribosyltransferases encoded by bacteriophage T4: catalytic properties and mutation analysis J Bacteriol 2004 186 7262 7272 10.1128/JB.186.21.7262-7272.2004 15489438 PMC523198

[B49] Wilkens K Tiemann B Bazan F Ruger W ADP-ribosylation and early transcription regulation by bacteriophage T4 Adv Exp Med Biol 1997 419 71 82 9193638 10.1007/978-1-4419-8632-0_8

[B50] Sommer N Salniene V Gineikiene E Nivinskas R Ruger W T4 early promoter strength probed in vivo with unribosylated and ADP-ribosylated Escherichia coli RNA polymerase: a mutation analysis Microbiology 2000 146 2643 2653 11021939 10.1099/00221287-146-10-2643

[B51] Kashlev M Nudler E Goldfarb A White T Kutter E Bacteriophage T4 Alc protein: a transcription termination factor sensing local modification of DNA Cell 1993 75 147 154 8402894

[B52] Severinov K Kashlev M Severinova E Bass I McWilliams K Kutter E Nikiforov V Snyder L Goldfarb A A non-essential domain of Escherichia coli RNA polymerase required for the action of the termination factor Alc J Biol Chem 1994 269 14254 14259 8188709

[B53] Tiemann B Depping R Ruger W Overexpression, purification, and partial characterization of ADP-ribosyltransferases modA and modB of bacteriophage T4 Gene Expr 1999 8 187 196 10634320 PMC6157369

[B54] Pene C Uzan M The bacteriophage T4 anti-sigma factor AsiA is not necessary for the inhibition of early promoters in vivo Mol Microbiol 2000 35 1180 1191 10.1046/j.1365-2958.2000.01787.x 10712698

[B55] Sanson B Uzan M Dual role of the sequence-specific bacteriophage T4 endoribonuclease RegB. mRNA inactivation and mRNA destabilization J Mol Biol 1993 233 429 446 10.1006/jmbi.1993.1522 8411154

[B56] Hirano N Ohshima H Takahashi H Biochemical analysis of the substrate specificity and sequence preference of endonuclease IV from bacteriophage T4, a dC-specific endonuclease implicated in restriction of dC-substituted T4 DNA synthesis Nucleic Acids Res 2006 34 4743 4751 10.1093/nar/gkl553 16971463 PMC1635256

[B57] Carlson K Lagerback P Nystrom AC Bacteriophage T4 endonuclease II: concerted single-strand nicks yield double-strand cleavage Mol Microbiol 2004 52 1403 1411 10.1111/j.1365-2958.2004.04062.x 15165242

[B58] Stevens A Rhoton JC Characterization of an inhibitor causing potassium chloride sensitivity of an RNA polymerase from T4 phage-infected Escherichia coli Biochemistry 1975 14 5074 5079 10.1021/bi00694a007 1103966

[B59] Orsini G Ouhammouch M Le Caer JP Brody EN The asiA gene of bacteriophage T4 codes for the anti-sigma 70 protein J Bacteriol 1993 175 85 93 8416914 10.1128/jb.175.1.85-93.1993 PMC196100

[B60] Adelman K Orsini G Kolb A Graziani L Brody EN The interaction between the AsiA protein of bacteriophage T4 and the sigma70 subunit of Escherichia coli RNA polymerase J Biol Chem 1997 272 27435 27443 10.1074/jbc.272.43.27435 9341196

[B61] Severinova E Severinov K Fenyo D Marr M Brody EN Roberts JW Chait BT Darst SA Domain organization of the Escherichia coli RNA polymerase sigma 70 subunit J Mol Biol 1996 263 637 647 10.1006/jmbi.1996.0604 8947564

[B62] Colland F Orsini G Brody EN Buc H Kolb A The bacteriophage T4 AsiA protein: a molecular switch for sigma 70-dependent promoters Mol Microbiol 1998 27 819 829 10.1046/j.1365-2958.1998.00729.x 9515707

[B63] Severinov K Muir TW Expressed protein ligation, a novel method for studying protein-protein interactions in transcription J Biol Chem 1998 273 16205 16209 10.1074/jbc.273.26.16205 9632677

[B64] Sharma UK Ravishankar S Shandil RK Praveen PV Balganesh TS Study of the interaction between bacteriophage T4 asiA and Escherichia coli sigma(70), using the yeast two-hybrid system: neutralization of asiA toxicity to E. coli cells by coexpression of a truncated sigma(70) fragment J Bacteriol 1999 181 5855 5859 10482532 10.1128/jb.181.18.5855-5859.1999 PMC94111

[B65] Urbauer JL Adelman K Urbauer RJ Simeonov MF Gilmore JM Zolkiewski M Brody EN Conserved regions 4.1 and 4.2 of sigma(70) constitute the recognition sites for the anti-sigma factor AsiA, and AsiA is a dimer free in solution J Biol Chem 2001 276 41128 41132 10.1074/jbc.M106400200 11518715

[B66] Pahari S Chatterji D Interaction of bacteriophage T4 AsiA protein with Escherichia coli sigma70 and its variant FEBS Lett 1997 411 60 62 10.1016/S0014-5793(97)00668-6 9247142

[B67] Simeonov MF Bieber Urbauer RJ Gilmore JM Adelman K Brody EN Niedziela-Majka A Minakhin L Heyduk T Urbauer JL Characterization of the interactions between the bacteriophage T4 AsiA protein and RNA polymerase Biochemistry 2003 42 7717 7726 10.1021/bi0340797 12820881

[B68] Sharma UK Chatterji D Both regions 4.1 and 4.2 of E. coli sigma(70) are together required for binding to bacteriophage T4 AsiA in vivo Gene 2006 376 133 143 10.1016/j.gene.2006.02.017 16545925

[B69] Minakhin L Niedziela-Majka A Kuznedelov K Adelman K Urbauer JL Heyduk T Severinov K Interaction of T4 AsiA with its target sites in the RNA polymerase sigma70 subunit leads to distinct and opposite effects on transcription J Mol Biol 2003 326 679 690 10.1016/S0022-2836(02)01442-0 12581632

[B70] Dove SL Hochschild A Bacterial two-hybrid analysis of interactions between region 4 of the sigma(70) subunit of RNA polymerase and the transcriptional regulators Rsd from Escherichia coli and AlgQ from Pseudomonas aeruginosa J Bacteriol 2001 183 6413 6421 10.1128/JB.183.21.6413-6421.2001 11591686 PMC100137

[B71] Urbauer JL Simeonov MF Urbauer RJ Adelman K Gilmore JM Brody EN Solution structure and stability of the anti-sigma factor AsiA: implications for novel functions Proc Natl Acad Sci USA 2002 99 1831 1835 10.1073/pnas.032464699 11830637 PMC122279

[B72] Pal D Vuthoori M Pande S Wheeler D Hinton DM Analysis of regions within the bacteriophage T4 AsiA protein involved in its binding to the sigma70 subunit of E. coli RNA polymerase and its role as a transcriptional inhibitor and co-activator J Mol Biol 2003 325 827 841 10.1016/S0022-2836(02)01307-4 12527294

[B73] Lambert LJ Schirf V Demeler B Cadene M Werner MH Flipping a genetic switch by subunit exchange Embo J 2001 20 7149 7159 10.1093/emboj/20.24.7149 11742991 PMC125793

[B74] Gilmore JM Bieber Urbauer RJ Minakhin L Akoyev V Zolkiewski M Severinov K Urbauer JL Determinants of Affinity and Activity of the Anti-Sigma Factor AsiA Biochemistry 2010 in press 20545305 10.1021/bi1002635 PMC2929534

[B75] Sharma UK Praveen PV Balganesh TS Mutational analysis of bacteriophage T4 AsiA: involvement of N- and C-terminal regions in binding to sigma(70) of Escherichia coli in vivo Gene 2002 295 125 134 10.1016/S0378-1119(02)00831-4 12242019

[B76] Stevens A Losick R, Chamberlin M A salt-promoted inhibitor of RNA polymerase isolated from T4 phage-infected E. coli RNA Polymerase 1976 Cold Spring Harbor, N. Y.: Cold Spring Harbor Laboratory 617 627

[B77] Baxter K Lee J Minakhin L Severinov K Hinton DM Mutational Analysis of sigma(70) Region 4 Needed for Appropriation by the Bacteriophage T4 Transcription Factors AsiA and MotA J Mol Biol 2006 363 931 944 10.1016/j.jmb.2006.08.074 16996538 PMC1698951

[B78] Minakhin L Camarero JA Holford M Parker C Muir TW Severinov K Mapping the molecular interface between the sigma(70) subunit of E. coli RNA polymerase and T4 AsiA J Mol Biol 2001 306 631 642 10.1006/jmbi.2001.4445 11243776

[B79] Gregory BD Nickels BE Garrity SJ Severinova E Minakhin L Urbauer RJ Urbauer JL Heyduk T Severinov K Hochschild A A regulator that inhibits transcription by targeting an intersubunit interaction of the RNA polymerase holoenzyme Proc Natl Acad Sci USA 2004 101 4554 4559 10.1073/pnas.0400923101 15070756 PMC384785

[B80] Severinova E Severinov K Darst SA Inhibition of Escherichia coli RNA polymerase by bacteriophage T4 AsiA J Mol Biol 1998 279 9 18 10.1006/jmbi.1998.1742 9636696

[B81] Leirmo S Harrison C Cayley DS Burgess RR Record MT Jr Replacement of potassium chloride by potassium glutamate dramatically enhances protein-DNA interactions in vitro Biochemistry 1987 26 2095 2101 10.1021/bi00382a006 2887198

[B82] Zou LL Richardson JP Enhancement of transcription termination factor rho activity with potassium glutamate J Biol Chem 1991 266 10201 10209 1709928

[B83] Orsini G Kolb A Buc H The Escherichia coli RNA polymerase.anti-sigma 70 AsiA complex utilizes alpha-carboxyl-terminal domain upstream promoter contacts to transcribe from a -10/-35 promoter J Biol Chem 2001 276 19812 19819 10.1074/jbc.M010105200 11278617

[B84] Orsini G Igonet S Pene C Sclavi B Buckle M Uzan M Kolb A Phage T4 early promoters are resistant to inhibition by the anti-sigma factor AsiA Mol Microbiol 2004 52 1013 1028 10.1111/j.1365-2958.2004.04038.x 15130121

[B85] Kolesky S Ouhammouch M Brody EN Geiduschek EP Sigma competition: the contest between bacteriophage T4 middle and late transcription J Mol Biol 1999 291 267 281 10.1006/jmbi.1999.2953 10438620

[B86] Mattson T Richardson J Goodin D Mutant of bacteriophage T4D affecting expression of many early genes Nature 1974 250 48 50 10.1038/250048a0 4601455

[B87] Mattson T Van Houwe G Epstein RH Isolation and characterization of conditional lethal mutations in the mot gene of bacteriophage T4 J Mol Biol 1978 126 551 570 10.1016/0022-2836(78)90058-X 745239

[B88] Uzan M Brody E Favre R Nucleotide sequence and control of transcription of the bacteriophage T4 motA regulatory gene Mol Microbiol 1990 4 1487 1496 10.1111/j.1365-2958.1990.tb02059.x 2287273

[B89] Hinton DM March-Amegadzie R Gerber JS Sharma M Characterization of pre-transcription complexes made at a bacteriophage T4 middle promoter: involvement of the T4 MotA activator and the T4 AsiA protein, a sigma 70 binding protein, in the formation of the open complex J Mol Biol 1996 256 235 248 10.1006/jmbi.1996.0082 8594193

[B90] March-Amegadzie R Hinton DM The bacteriophage T4 middle promoter PuvsX: analysis of regions important for binding of the T4 transcriptional activator MotA and for activation of transcription Mol Microbiol 1995 15 649 660 10.1111/j.1365-2958.1995.tb02374.x 7783637

[B91] Stoskiene G Truncaite L Zajanckauskaite A Nivinskas R Middle promoters constitute the most abundant and diverse class of promoters in bacteriophage T4 Mol Microbiol 2007 64 421 434 10.1111/j.1365-2958.2007.05659.x 17371501

[B92] Cicero MP Alexander KA Kreuzer KN The MotA transcriptional activator of bacteriophage T4 binds to its specific DNA site as a monomer Biochemistry 1998 37 4977 4984 10.1021/bi972337s 9538016

[B93] Li N Sickmier EA Zhang R Joachimiak A White SW The MotA transcription factor from bacteriophage T4 contains a novel DNA-binding domain: the 'double wing' motif Mol Microbiol 2002 43 1079 1088 10.1046/j.1365-2958.2002.02809.x 11918797

[B94] Li N Zhang W White SW Kriwacki RW Solution structure of the transcriptional activation domain of the bacteriophage T4 protein, MotA Biochemistry 2001 40 4293 4302 10.1021/bi0028284 11284685

[B95] Finnin MS Hoffman DW Kreuzer KN Porter SJ Schmidt RP White SW The MotA protein from bacteriophage T4 contains two domains. Preliminary structural analysis by X-ray diffraction and nuclear magnetic resonance J Mol Biol 1993 232 301 304 10.1006/jmbi.1993.1384 8331666

[B96] Gerber JS Hinton DM An N-terminal mutation in the bacteriophage T4 motA gene yields a protein that binds DNA but is defective for activation of transcription J Bacteriol 1996 178 6133 6139 8892810 10.1128/jb.178.21.6133-6139.1996 PMC178481

[B97] Pande S Makela A Dove SL Nickels BE Hochschild A Hinton DM The bacteriophage T4 transcription activator MotA interacts with the far-C-terminal region of the sigma70 subunit of Escherichia coli RNA polymerase J Bacteriol 2002 184 3957 3964 10.1128/JB.184.14.3957-3964.2002 12081968 PMC135182

[B98] Finnin MS Cicero MP Davies C Porter SJ White SW Kreuzer KN The activation domain of the MotA transcription factor from bacteriophage T4 Embo J 1997 16 1992 2003 10.1093/emboj/16.8.1992 9155025 PMC1169802

[B99] Guild N Gayle M Sweeney R Hollingsworth T Modeer T Gold L Transcriptional activation of bacteriophage T4 middle promoters by the motA protein J Mol Biol 1988 199 241 258 10.1016/0022-2836(88)90311-7 3280803

[B100] Marshall P Sharma M Hinton DM The bacteriophage T4 transcriptional activator MotA accepts various base-pair changes within its binding sequence J Mol Biol 1999 285 931 944 10.1006/jmbi.1998.2373 9918715

[B101] Truncaite L Piesiniene L Kolesinskiene G Zajanckauskaite A Driukas A Klausa V Nivinskas R Twelve new MotA-dependent middle promoters of bacteriophage T4: consensus sequence revised J Mol Biol 2003 327 335 346 10.1016/S0022-2836(03)00125-6 12628241

[B102] Truncaite L Zajanckauskaite A Nivinskas R Identification of two middle promoters upstream DNA ligase gene 30 of bacteriophage T4 J Mol Biol 2002 317 179 190 10.1006/jmbi.2002.5407 11902835

[B103] Sharma M Marshall P Hinton DM Binding of the bacteriophage T4 transcriptional activator, MotA, to T4 middle promoter DNA: evidence for both major and minor groove contacts J Mol Biol 1999 290 905 915 10.1006/jmbi.1999.2928 10438591

[B104] Bonocora RP Caignan G Woodrell C Werner MH Hinton DM A basic/hydrophobic cleft of the T4 activator MotA interacts with the C-terminus of E. coli sigma70 to activate middle gene transcription Mol Microbiol 2008 69 331 343 10.1111/j.1365-2958.2008.06276.x 18485078 PMC2631437

[B105] Kuldell N Hochschild A Amino acid substitutions in the -35 recognition motif of sigma 70 that result in defects in phage lambda repressor-stimulated transcription J Bacteriol 1994 176 2991 2998 8188599 10.1128/jb.176.10.2991-2998.1994 PMC205456

[B106] Li M Moyle H Susskind MM Target of the transcriptional activation function of phage lambda cI protein Science 1994 263 75 77 10.1126/science.8272867 8272867

[B107] Nickels BE Dove SL Murakami KS Darst SA Hochschild A Protein-protein and protein-DNA interactions of sigma70 region 4 involved in transcription activation by lambda cI J Mol Biol 2002 324 17 34 10.1016/S0022-2836(02)01043-4 12421556

[B108] Rhodius VA Busby SJ Interactions between activating region 3 of the Escherichia coli cyclic AMP receptor protein and region 4 of the RNA polymerase sigma(70) subunit: application of suppression genetics J Mol Biol 2000 299 311 324 10.1006/jmbi.2000.3737 10860740

[B109] Lonetto MA Rhodius V Lamberg K Kiley P Busby S Gross C Identification of a contact site for different transcription activators in region 4 of the Escherichia coli RNA polymerase sigma70 subunit J Mol Biol 1998 284 1353 1365 10.1006/jmbi.1998.2268 9878355

[B110] Landini P Busby SJ The Escherichia coli Ada protein can interact with two distinct determinants in the sigma70 subunit of RNA polymerase according to promoter architecture: identification of the target of Ada activation at the alkA promoter J Bacteriol 1999 181 1524 1529 10049384 10.1128/jb.181.5.1524-1529.1999 PMC93542

[B111] Bhende PM Egan SM Genetic evidence that transcription activation by RhaS involves specific amino acid contacts with sigma 70 J Bacteriol 2000 182 4959 4969 10.1128/JB.182.17.4959-4969.2000 10940041 PMC111377

[B112] Wickstrum JR Egan SM Amino acid contacts between sigma 70 domain 4 and the transcription activators RhaS and RhaR J Bacteriol 2004 186 6277 6285 10.1128/JB.186.18.6277-6285.2004 15342598 PMC515164

[B113] Decker KB Hinton DM The secret to 6S: regulating RNA polymerase by ribo-sequestration Mol Microbiol 2009 73 137 140 10.1111/j.1365-2958.2009.06759.x 19538446 PMC3111051

[B114] Pulitzer JF Coppo A Caruso M Host--virus interactions in the control of T4 prereplicative transcription. II. Interaction between tabC (rho) mutants and T4 mot mutants J Mol Biol 1979 135 979 997 10.1016/0022-2836(79)90523-0 395323

[B115] Cicero MP Sharp MM Gross CA Kreuzer KN Substitutions in bacteriophage T4 AsiA and Escherichia coli sigma(70) that suppress T4 motA activation mutations J Bacteriol 2001 183 2289 2297 10.1128/JB.183.7.2289-2297.2001 11244069 PMC95136

[B116] Yuan AH Nickels BE Hochschild A The bacteriophage T4 AsiA protein contacts the beta-flap domain of RNA polymerase Proc Natl Acad Sci USA 2009 106 6597 6602 10.1073/pnas.0812832106 19366670 PMC2672554

[B117] Yuan AH Hochschild A Direct activator/co-activator interaction is essential for bacteriophage T4 middle gene expression Mol Microbiol 2009 74 1018 1030 10.1111/j.1365-2958.2009.06916.x 19843221 PMC5673250

[B118] Yuan AH Hochschild A Direct activator/co-activator interaction is essential for bacteriophage T4 middle gene expression Mol Microbiol 2009 19843221 10.1111/j.1365-2958.2009.06916.x PMC5673250

[B119] Pineda M Gregory BD Szczypinski B Baxter KR Hochschild A Miller ES Hinton DM A family of anti-sigma70 proteins in T4-type phages and bacteria that are similar to AsiA, a Transcription inhibitor and co-activator of bacteriophage T4 J Mol Biol 2004 344 1183 1197 10.1016/j.jmb.2004.10.003 15561138

[B120] James TD Cashel M Hinton DM A mutation within the β subunit of Escherichia coli RNA polymerase impairs transcription from bacteriophage T4 middle promoters J Bacteriol 2010 in press 20729353 10.1128/JB.00338-10 PMC2953677

[B121] Adelman K Brody EN Buckle M Stimulation of bacteriophage T4 middle transcription by the T4 proteins MotA and AsiA occurs at two distinct steps in the transcription cycle Proc Natl Acad Sci USA 1998 95 15247 15252 10.1073/pnas.95.26.15247 9860954 PMC28028

[B122] Hinton DM Vuthoori S Efficient inhibition of Escherichia coli RNA polymerase by the bacteriophage T4 AsiA protein requires that AsiA binds first to free sigma70 J Mol Biol 2000 304 731 739 11188759 10.1006/jmbi.2000.4113

[B123] Wade JT Struhl K Association of RNA polymerase with transcribed regions in Escherichia coli Proc Natl Acad Sci USA 2004 101 17777 17782 10.1073/pnas.0404305101 15596728 PMC539717

[B124] Miller ES Heidelberg JF Eisen JA Nelson WC Durkin AS Ciecko A Feldblyum TV White O Paulsen IT Nierman WC Complete genome sequence of the broad-host-range vibriophage KVP40: comparative genomics of a T4-related bacteriophage J Bacteriol 2003 185 5220 5233 10.1128/JB.185.17.5220-5233.2003 12923095 PMC180978

[B125] Rao X Deighan P Hua Z Hu X Wang J Luo M Liang Y Zhong G Hochschild A Shen L A regulator from Chlamydia trachomatis modulates the activity of RNA polymerase through direct interaction with the beta subunit and the primary sigma subunit Genes Dev 2009 23 1818 1829 10.1101/gad.1784009 19651989 PMC2720258

[B126] Menkens AE Kreuzer KN Deletion analysis of bacteriophage T4 tertiary origins. A promoter sequence is required for a rifampicin-resistant replication origin J Biol Chem 1988 263 11358 11365 3403531

[B127] Benson KH Kreuzer KN Role of MotA transcription factor in bacteriophage T4 DNA replication J Mol Biol 1992 228 88 100 10.1016/0022-2836(92)90493-4 1447797

[B128] Luke K Radek A Liu X Campbell J Uzan M Haselkorn R Kogan Y Microarray analysis of gene expression during bacteriophage T4 infection Virology 2002 299 182 191 10.1006/viro.2002.1409 12202221

[B129] Carpousis AJ Mudd EA Krisch HM Transcription and messenger RNA processing upstream of bacteriophage T4 gene 32 Mol Gen Genet 1989 219 39 48 10.1007/BF00261155 2615764

[B130] Sanson B Uzan M Sequence and characterization of the bacteriophage T4 comC alpha gene product, a possible transcription antitermination factor J Bacteriol 1992 174 6539 6547 1400206 10.1128/jb.174.20.6539-6547.1992 PMC207620

[B131] Chiurazzi M Pulitzer JF Characterisation of the bacteriophage T4 comC alpha 55.6 and comCJ mutants. A possible role in an antitermination process FEMS Microbiol Lett 1998 166 187 195 9770273 10.1111/j.1574-6968.1998.tb13889.x

[B132] Dodd IB Shearwin KE Egan JB Revisited gene regulation in bacteriophage lambda Curr Opin Genet Dev 2005 15 145 152 10.1016/j.gde.2005.02.001 15797197

[B133] Washburn RS Stitt BL In vitro characterization of transcription termination factor Rho from Escherichia coli rho(nusD) mutants J Mol Biol 1996 260 332 346 10.1006/jmbi.1996.0404 8757797

[B134] Sozhamannan S Stitt BL Effects on mRNA degradation by Escherichia coli transcription termination factor Rho and pBR322 copy number control protein Rop J Mol Biol 1997 268 689 703 10.1006/jmbi.1997.1004 9175854

[B135] Glisovic T Bachorik JL Yong J Dreyfuss G RNA-binding proteins and post-transcriptional gene regulation FEBS Lett 2008 582 1977 1986 10.1016/j.febslet.2008.03.004 18342629 PMC2858862

[B136] Zhang Z Klatt A Henderson AJ Gilmour DS Transcription termination factor Pcf11 limits the processivity of Pol II on an HIV provirus to repress gene expression Genes Dev 2007 21 1609 1614 10.1101/gad.1542707 17606639 PMC1899470

[B137] Ebright RH RNA polymerase: structural similarities between bacterial RNA polymerase and eukaryotic RNA polymerase II J Mol Biol 2000 304 687 698 10.1006/jmbi.2000.4309 11124018

[B138] Werner F Structural evolution of multisubunit RNA polymerases Trends Microbiol 2008 16 247 250 10.1016/j.tim.2008.03.008 18468900

[B139] Freiman RN Albright SR Zheng S Sha WC Hammer RE Tjian R Requirement of tissue-selective TBP-associated factor TAFII105 in ovarian development Science 2001 293 2084 2087 10.1126/science.1061935 11557891

[B140] Isogai Y Keles S Prestel M Hochheimer A Tjian R Transcription of histone gene cluster by differential core-promoter factors Genes Dev 2007 21 2936 2949 10.1101/gad.1608807 17978101 PMC2049195

[B141] Daegelen P Brody E The rIIA gene of bacteriophage T4. II. Regulation of its messenger RNA synthesis Genetics 1990 125 249 260 2379818 10.1093/genetics/125.2.249 PMC1204015

[B142] Daegelen P Brody E The rIIA gene of bacteriophage T4. I. Its DNA sequence and discovery of a new open reading frame between genes 60 and rIIA Genetics 1990 125 237 248 2379817 10.1093/genetics/125.2.237 PMC1204014

[B143] Hsu T Karam JD Transcriptional mapping of a DNA replication gene cluster in bacteriophage T4. Sites for initiation, termination, and mRNA processing J Biol Chem 1990 265 5303 5316 2180963

[B144] Barth KA Powell D Trupin M Mosig G Regulation of two nested proteins from gene 49 (recombination endonuclease VII) and of a lambda RexA-like protein of bacteriophage T4 Genetics 1988 120 329 343 2974005 10.1093/genetics/120.2.329 PMC1203513

[B145] Gruidl ME Mosig G Sequence and transcripts of the bacteriophage T4 DNA repair gene uvsY Genetics 1986 114 1061 1079 3026891 10.1093/genetics/114.4.1061 PMC1203028

[B146] Nivinskas RG Raudonikene AA Guild N A new early gene in front of the middle gene 31 of bacteriophage T4: cloning and expression Mol Biol (Mosk) 1989 23 739 749 2671675

[B147] Gott JM Zeeh A Bell-Pedersen D Ehrenman K Belfort M Shub DA Genes within genes: independent expression of phage T4 intron open reading frames and the genes in which they reside Genes Dev 1988 2 1791 1799 10.1101/gad.2.12b.1791 2467840

[B148] Tseng MJ He P Hilfinger JM Greenberg GR Bacteriophage T4 nrdA and nrdB genes, encoding ribonucleotide reductase, are expressed both separately and coordinately: characterization of the nrdB promoter J Bacteriol 1990 172 6323 6332 2228963 10.1128/jb.172.11.6323-6332.1990 PMC526816

[B149] Belin D Mudd EA Prentki P Yi-Yi Y Krisch HM Sense and antisense transcription of bacteriophage T4 gene 32. Processing and stability of the mRNAs J Mol Biol 1987 194 231 243 10.1016/0022-2836(87)90371-8 3612804

[B150] Estrem ST Ross W Gaal T Chen ZW Niu W Ebright RH Gourse RL Bacterial promoter architecture: subsite structure of UP elements and interactions with the carboxy-terminal domain of the RNA polymerase alpha subunit Genes Dev 1999 13 2134 2147 10.1101/gad.13.16.2134 10465790 PMC316962

